# Supplementary material for: Use of Health Services and Support Resources by Immediate Family Members Bereaved by Suicide: A Scoping Review
Source: Int J Environ Res Public Health. 2022 Aug 14;19(16):10016. doi: 10.3390/ijerph191610016 (PMC9408753; doi:10.3390/ijerph191610016)
Supplement: Supplementary file 1 [file ijerph-19-10016-s001.zip › ijerph-1832255-supplementary/ijerph-1832255-supplementary/Supplementary File S1.pdf]

Supplementary file

Example of search strategies in Ovid (APA PsycInfo, Embase and Medline)
25.6.2020 Ovid: Abstract Reference
ovidsp.dc1.ovid.com/sp-4.06.0a/ovidweb.cgi 1/268

#	Searches	Results
1	"Health care".mp. [mp=ti, ab, hw, tc, id, ot, tm, mh, tn, dm, mf, dv, kw, fx, dq, nm, kf, ox, px, rx, ui, sy]	2541005
2	Healthcare.mp. [mp=ti, ab, hw, tc, id, ot, tm, mh, tn, dm, mf, dv, kw, fx, dq, nm, kf, ox, px, rx, ui, sy]	675417
3	outpatient.mp. [mp=ti, ab, hw, tc, id, ot, tm, mh, tn, dm, mf, dv, kw, fx, dq, nm, kf, ox, px, rx, ui, sy]	475591
4	inpatient.mp. [mp=ti, ab, hw, tc, id, ot, tm, mh, tn, dm, mf, dv, kw, fx, dq, nm, kf, ox, px, rx, ui, sy]	245479
5	treat*.mp. [mp=ti, ab, hw, tc, id, ot, tm, mh, tn, dm, mf, dv, kw, fx, dq, nm, kf, ox, px, rx, ui, sy]	14981026
6	support*.mp. [mp=ti, ab, hw, tc, id, ot, tm, mh, tn, dm, mf, dv, kw, fx, dq, nm, kf, ox, px, rx, ui, sy]	12704422
7	exp "Delivery of Health Care"/	4335030
8	exp Health Services/	7519555
9	exp Primary Health Care/	345170
10	exp Secondary Care/	8262
11	exp Community Health Services/	417651
12	exp Mental Health Services/	191667
13	exp Community Mental Health Services/	26370
14	exp Outpatients/	145098
15	exp Inpatients/	197027
16	"follow up".mp.	3411437
17	follow-up.mp.	3411437
18	exp Suicide/	151464
19	Suicid*.mp. [mp=ti, ab, hw, tc, id, ot, tm, mh, tn, dm, mf, dv, kw, fx, dq, nm, kf, ox, px, rx, ui, sy]	293570
20	exp Bereavement/	36817
21	bereave*.mp. [mp=ti, ab, hw, tc, id, ot, tm, mh, tn, dm, mf, dv, kw, fx, dq, nm, kf, ox, px, rx, ui, sy]	35268
22	exp Grief/	34293
23	Grie*.mp. [mp=ti, ab, hw, tc, id, ot, tm, mh, tn, dm, mf, dv, kw, fx, dq, nm, kf, ox, px, rx, ui, sy]	60944
24	Mourn*.mp. [mp=ti, ab, hw, tc, id, ot, tm, mh, tn, dm, mf, dv, kw, fx, dq, nm, kf, ox, px, rx, ui, sy]	10666
25	exp Counseling/	286650
26	counsel*.mp. [mp=ti, ab, hw, tc, id, ot, tm, mh, tn, dm, mf, dv, kw, fx, dq, nm, kf, ox, px, rx, ui, sy]	480530
27	exp "Referral and Consultation"/	187804
28	Consult*.mp. [mp=ti, ab, hw, tc, id, ot, tm, mh, tn, dm, mf, dv, kw, fx, dq, nm, kf, ox, px, rx, ui, sy]	476038
29	team*.mp. [mp=ti, ab, hw, tc, id, ot, tm, mh, tn, dm, mf, dv, kw, fx, dq, nm, kf, ox, px, rx, ui, sy]	562161
30	exp Hospitals/	1416912
31	hospital*.mp. [mp=ti, ab, hw, tc, id, ot, tm, mh, tn, dm, mf, dv, kw, fx, dq, nm, kf, ox, px, rx, ui, sy]	4315175
32	exp Ambulatory Care Facilities/ or exp Ambulatory Care/	220226
33	ambulatory*.mp. [mp=ti, ab, hw, tc, id, ot, tm, mh, tn, dm, mf, dv, kw, fx, dq, nm, kf, ox, px, rx, ui, sy]	327785
34	("Health service" or "Health services" or "Health care service" or "Health care services" or "Healthcare service" or "Healthcare services" or "Medical health care" or "Medical healthcare" or "Medical care" or "Primary health care" or "Primary healthcare" or "Primary care" or "Secondary health care" or "Secondary healthcare" or "Secondary care" or "Specialist health care" or "Specialist healthcare" or "Specialist care" or "Specialized health care" or "Specialized healthcare" or "Specialized care" or "Extramural health care" or "Extramural healthcare" or "Extramural care" or "Mental health care" or "Mental healthcare" or "Mental care" or "Community health care" or "Community healthcare" or "Community care" or "Community mental health care" or "Community mental healthcare" or "Community mental care").mp. [mp=ti, ab, hw, tc, id, ot, tm, mh, tn, dm, mf, dv, kw, fx, dq, nm, kf, ox, px, rx, ui, sy]	1696504
35	18 or 19	293570
36	20 or 21 or 22 or 23 or 24	84 618
37	7 or 8 or 9 or 10 or 11 or 12 or 13 or 14 or 15 or 25 or 27 or 30 or 32	9239099
38	1 or 2 or 3 or 4 or 5 or 6 or 16 or 17 or 26 or 28 or 29 or 31 or 33 or 34	30657371
39	37 or 38	32642891
40	postvention.mp.	751
41	39 or 40	32643096
42	35 and 36 and 41	3840
43	limit 42 to yr="2010 -Current"	1977
