# Supplementary material for: Use of Health Services and Support Resources by Immediate Family Members Bereaved by Suicide: A Scoping Review
Source: Int J Environ Res Public Health. 2022 Aug 14;19(16):10016. doi: 10.3390/ijerph191610016 (PMC9408753; doi:10.3390/ijerph191610016)
Supplement: Supplementary file 1 [file ijerph-19-10016-s001.zip › ijerph-1832255-supplementary/ijerph-1832255-supplementary/Supplementary File S3_NetworkAnalysis.pdf]

Supplementary File S3: Network analysis

To do a network analysis of co-publications, we applied VOSviewer, a free software tool for constructing and visualizing bibliometric networks, on the 63 included studies in our scoping review. A librarian at SINTEF conducted the network analysis. All references were searched in the abstract and citation database Scopus, to create the necessary CSV-file needed for VOSviewer. Except one manual adjustment of author affiliation for a Danish study (Erlangsen et al. 2021), all raw data come from Scopus.

Figure S1 is based on information on the author's affiliations and shows co-authorship across all publications, independently of the number of publications each author has contributed in. The size of the bullets indicates number of publications, while the colour of the bullets indicates year of publication. The line/link between the bullets indicates co-authorship between countries. For example, Dr. David Gunnel from Bristol University contributed in the Danish study of Erlangsen et al. (2021), indicated by a link between UK and Denmark. As described in our main manuscript, Australia, US and UK account for more than half of the 63 publications included in our review.

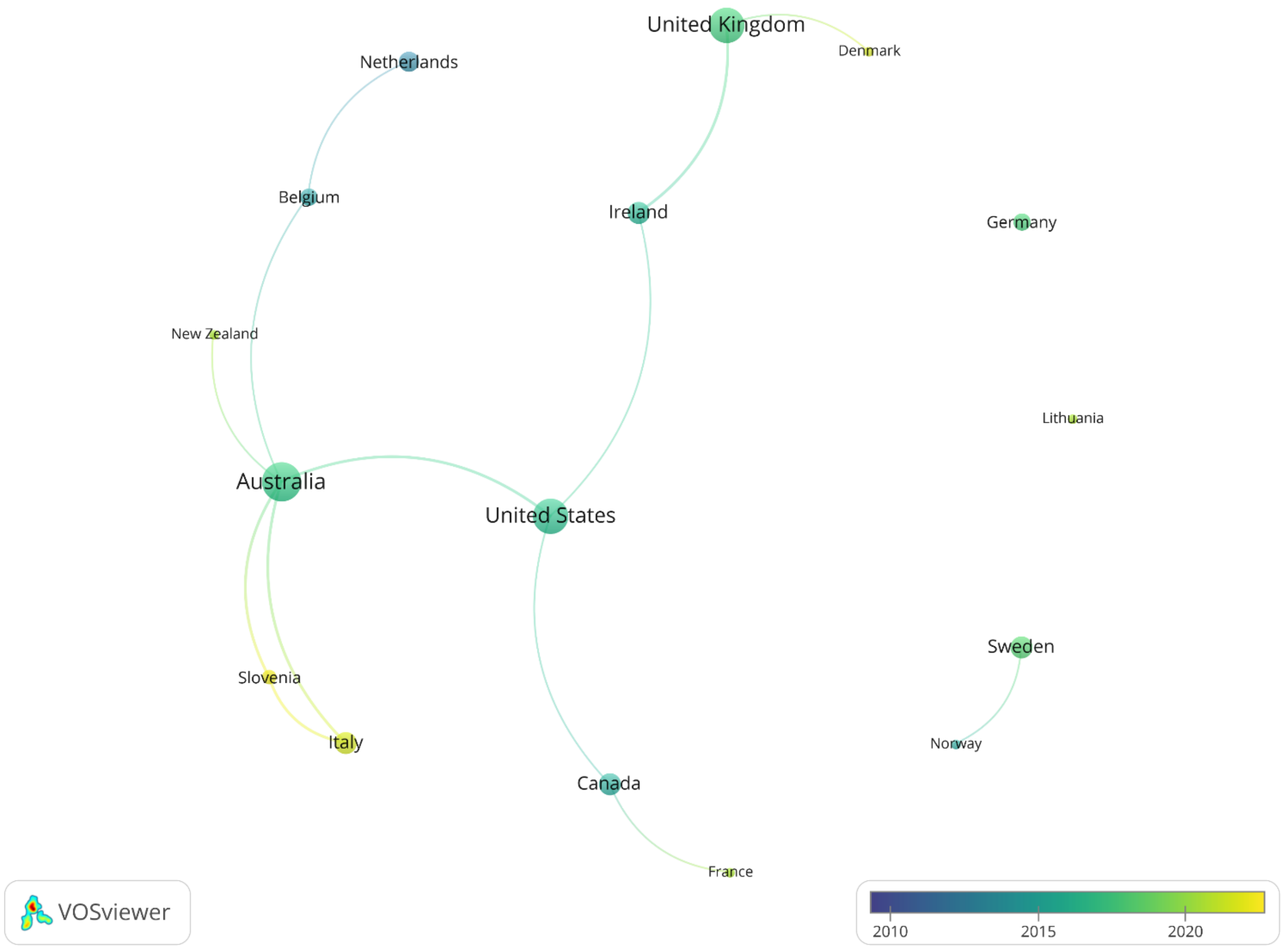

Figure S1 Network analysis based on country. Year of publication 2010-2022.

Figure S1 was based on the data presented in Table S1:

Table S1 Data in network analysis based on country.

Selected	Country	Documents	Citations	Total link strength
☑	australia	16	195	8
☑	italy	5	16	4
☑	slovenia	2	4	4
☑	united states	13	143	4
☑	ireland	5	53	3
☑	united kingdom	13	152	3
☑	belgium	3	64	2
☑	canada	5	63	2
☑	denmark	1	0	1
☑	france	1	5	1
☑	netherlands	4	108	1
☑	new zealand	1	3	1
☑	norway	1	5	1
☑	sweden	5	26	1
☑	germany	3	25	0
☑	lithuania	1	1	0

In Figure S2 we present an analysis of the number of citations within and between authors in the 63 included articles. The size of the bullets indicates number of total citations (including other studies not included in this review), the lines indicate who cited who within the included articles in the review. Colour of the bullets indicates year of publication.

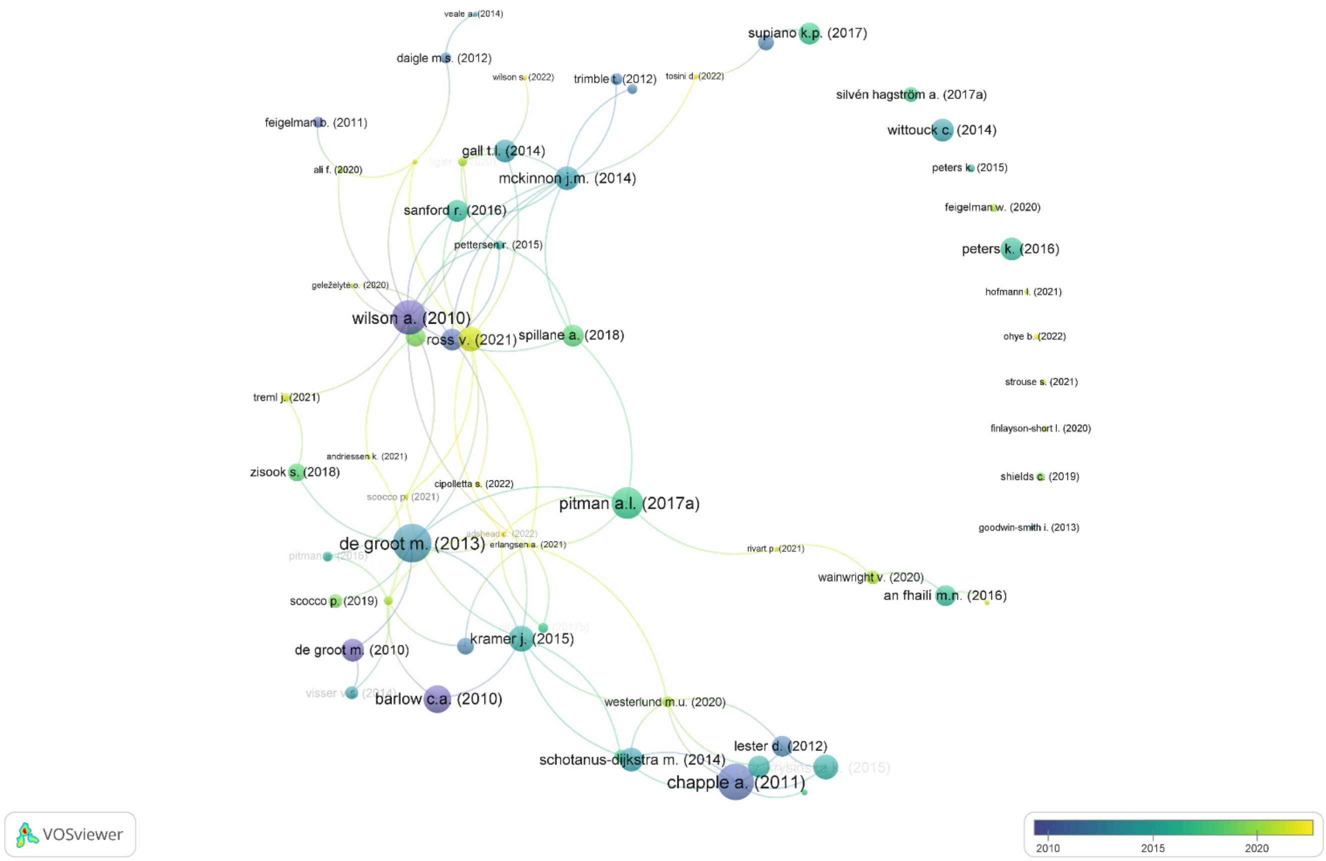

Figure S2 Citation analysis. Year of publication 2010-2022.

Figure S2 was based on the data presented in Table S2:

Table S2 Data in citation analysis.

Selected	Document	Citations	Links
☑	wilson a. (2010)	37	11
☑	mckinnon j.m. (2014)	21	9
☑	ross v. (2021)	23	8
☑	kramer j. (2015)	24	7
☑	de groot m. (2013)	43	7
☑	erlangsen a. (2021)	0	6
☑	gehrmann m. (2020)	5	6
☑	westerlund m.u. (2020)	7	6
☑	chapple a. (2011)	41	6
☑	adshead c. (2022)	0	5
☑	pitman a.l. (2017a)	32	5
☑	schneider b. (2011)	18	5
☑	silvén hagström a. (2021)	0	4
☑	scocco p. (2021)	2	4
☑	ligier f. (2020)	5	4
☑	andriessen k. (2019)	16	4
☑	spillane a. (2018)	18	4
☑	pettersen r. (2015)	5	4
☑	schotanus-dijkstra m. (2014)	21	4
☑	ali f. (2020)	3	3
☑	bailey e. (2017)	9	3
☑	sanford r. (2016)	19	3
☑	bailey l. (2015)	19	3
☑	gall t.l. (2014)	20	3
☑	lester d. (2012)	17	3
☑	cipolletta s. (2022)	3	2
☑	tosini d. (2022)	0	2
☑	rivart p. (2021)	0	2
☑	andriessen k. (2021)	0	2
☑	treml j. (2021)	5	2
☑	geležėlytė o. (2020)	1	2
☑	wainwright v. (2020)	10	2
☑	zisook s. (2018)	14	2
☑	pitman a.l. (2017b)	6	2
☑	pitman a. (2016)	6	2
☑	an fhailí m.n. (2016)	17	2
☑	krysinska k. (2015)	23	2
☑	visser v.s. (2014)	9	2
☑	daigle m.s. (2012)	7	2
☑	supiano k.p. (2012)	12	2
☑	hawton k. (2012)	13	2
☑	barlow c.a. (2010)	27	2
☑	de groot m. (2010)	20	2
☑	wilson s. (2022)	1	1
☑	entilli l. (2021)	1	1
☑	scocco p. (2019)	10	1
☑	supiano k.p. (2017)	18	1
☑	silvén hagström a. (2017b)	3	1
☑	veale a. (2014)	0	1
☑	trimble t. (2012)	8	1
☑	miers d. (2012)	6	1
☑	feigelman b. (2011)	7	1
☑	ohye b. (2022)	3	0
☑	strouse s. (2021)	3	0
☑	hofmann l. (2021)	2	0
☑	feigelman w. (2020)	4	0
☑	finlayson-short l. (2020)	3	0
☑	shields c. (2019)	5	0
☑	silvén hagström a. (2017a)	11	0
☑	peters k. (2016)	20	0
☑	peters k. (2015)	4	0
☑	wittouck c. (2014)	20	0
☑	goodwin-smith i. (2013)	2	0
